# What to Observe at the Bed-side and after Death

**Published:** 1855-03

**Authors:** 


**WHAT TO OBSERVE AT THE BED-SIDE AND AFTER DEATH IN MEDICAL** Cases. Published under the Authority of the London Society of Observation. Philadelphia: Blanchard & Lea, pp. 228.

We have sometimes thought that, if there be a prominent error in our Medical literature, it may be that of telling too much. Undoubtedly a superfluity of expression is an error. The age wreaks itself on language. It seems a dull condition, where nothing is left unsaid. We cannot endure to have everything explained, and illustrated and amplified, the passive physician being all the while a dull recipient of this inpouring. Here, however, is a work which is happily suggestive. With its closely compact and abbreviated fragments of sentences, it leaves much to be said and more to be thought of. No man who accustoms himself to the substantial comfort of doing his own thinking, can use this book without eminent advantage. While it leaves so much unsaid, it leaves nothing unsuggested. Taking up the several structures and organs of the system, it closely scrutinizes each with reference to its manifold phenomena of disease. We think our readers who read this book judiciously, cannot fail to derive signal benefit from it. It is convenient in size, and may be readily ordered of the Publishers, and received by mail.

The Medical Counsellor, is the title of a new Medical Journal, published *weekly*, at Columbus, Ohio, and edited by R. Hills, M. D. This is the second Weekly Medical Journal published in this country, the other being the Boston Medical Journal; and neither of these is by any means *weakly*. The editor and proprietor must calculate upon a stupendous circulation to publish his journal so cheaply, and yet it seems cheap *only* in the matter of price. We hope the Counsellor may have a wide range of consultation practice, and we shall certainly be glad to exchange professional opinions with him.

